# An Artificial Intelligence Model for ECG-Based Prediction of Heart Failure with Preserved Ejection Fraction Diagnosis

**DOI:** 10.3390/jcdd13070340

**Published:** 2026-07-21

**Authors:** Ibrahim Karabayir, Shekhar Singh, Tolga Hayit, Elsayed Z. Soliman, Dalane W. Kitzman, David M. Herrington, Barry A. Borlaug, Robert L. Davis, Sanjiv Shah, Oguz Akbilgic

**Affiliations:** 1 Department of Cardiovascular Medicine, Wake Forest School of Medicine, Winston-Salem, NC 27157, USA; 2 Department of Cardiovascular Medicine, Mayo Clinic, Rochester, MN 55905, USA; 3 Center for Biomedical Informatics, University of Tennessee Health Science Center, Memphis, TN 38103, USA; 4 Division of Cardiology, Northwestern University Feinberg School of Medicine, Chicago, IL 60611, USA

**Keywords:** ECG-AI, HFpEF, artificial intelligence, heart failure, risk prediction, electrocardiogram, deep learning

## Abstract

Background: Heart failure with preserved ejection fraction (HFpEF) accounts for over half of all heart failure (HF) cases and remains diagnostically challenging due to nonspecific symptoms and a lack of accessible noninvasive screening tools, leading to widespread underdiagnosis and delayed treatment. The electrocardiogram (ECG) is a low-cost, widely available tool that reflects myocardial electrical remodeling. We previously developed and externally validated an ECG-based artificial intelligence (ECG-AI) model capable of classifying ECGs into four categories: reduced ejection fraction (rEF), mid-range ejection fraction (mEF), HFpEF, and controls. In this study, we evaluate the ability of this ECG-AI model to predict future HFpEF diagnosis using real-world data from a large integrated health system. Methods: We applied the validated ECG-AI model to an independent cohort of 7713 patients from Wake Forest Baptist Health (WFBH), using one ECG per patient recorded before the clinical diagnosis of HFpEF. Model discrimination was evaluated across multiple prediction windows from six months up to ten years before diagnosis using the area under the receiver operating characteristic curve (AUC), with comparisons performed using DeLong’s test. Performance was also compared head-to-head with the H2FPEF score, a validated clinical score for HFpEF, in the subset of patients with complete data for score calculation. ECG-AI outputs were stratified into quartiles and evaluated using Kaplan–Meier survival analysis and multivariable Cox proportional hazards regression adjusted for demographics and major comorbidities. Results: Of the 7713 patients, 283 (3.7%) were subsequently diagnosed with HFpEF. The ECG-AI model achieved an AUC of 0.79 (95% CI, 0.72–0.86) using ECGs recorded within six months before diagnosis. Discrimination remained stable across prediction windows extending up to ten years before diagnosis (AUC range, 0.78–0.80; all DeLong *p* > 0.01 vs. the six-month window). Compared with the H2FPEF score, ECG-AI demonstrated superior discrimination, improving the AUC by 0.06–0.07 across the evaluated prediction windows. Patients in the highest ECG-AI risk quartile had an unadjusted hazard ratio (HR) of 11.18 (95% CI, 7.27–17.21; C-index, 0.75) and an adjusted HR of 6.60 (95% CI, 4.24–10.28; C-index, 0.82). Adding clinical covariates to ECG-AI improved the C-index by 0.07 and 0.09 for the five- and ten-year prediction windows, respectively. Conclusions: An independently validated ECG-AI model predicted incident HFpEF up to ten years before clinical diagnosis and outperformed the H2FPEF score using ECG alone, indicating that ECG-derived signatures precede clinical recognition of the syndrome. ECG-AI shows promise as a prognostic, risk-stratification tool to prioritize further evaluation; prospective validation and single-lead assessment are needed before screening or wearable deployment.

## 1. Introduction

Heart failure (HF) affects approximately 6.5 million Americans and over 64 million individuals globally, with US prevalence projected to exceed 8 million by 2030 [1,2,3,4]. HF imposes a substantial clinical and economic burden, and age-adjusted HF mortality has been increasing since 2012 [1,2]. Among HF subtypes, heart failure with preserved ejection fraction (HFpEF)—defined by HF symptoms in the presence of a left ventricular ejection fraction (LVEF) ≥50%—now accounts for more than half of all HF cases worldwide and is projected to become the dominant HF phenotype as the population ages [5,6,7].

Despite its growing prevalence, HFpEF remains one of the most diagnostically challenging cardiovascular conditions. Unlike HF with reduced ejection fraction (HFrEF), which can be reliably identified by noninvasive echocardiographic measurement of LVEF, HFpEF lacks a similarly accessible diagnostic biomarker. The clinical gold standard—invasive hemodynamic exercise testing demonstrating elevated pulmonary capillary wedge pressure—is resource-intensive and impractical for widespread screening [8,9,10]. Noninvasive scoring systems, including the H2FPEF score and HFA-PEFF diagnostic algorithm, offer alternative approaches but are limited by imperfect sensitivity and specificity, particularly in patients with multiple comorbidities [11,12,13,14,15].

The presenting symptoms of HFpEF—exertional dyspnea, fatigue, and reduced exercise tolerance—are nonspecific and frequently attributed to obesity, deconditioning, or pulmonary disease [16,17]. This diagnostic ambiguity, combined with heterogeneous underlying pathophysiology, contributes to frequent misdiagnosis and delayed therapy [5,6,7]. Unlike HFrEF, HFpEF hospitalizations are rising, with longer hospital stays, worse outcomes, and greater costs [5,18,19]. These considerations highlight an urgent unmet need for low-cost, scalable, noninvasive tools capable of identifying individuals at elevated risk for HFpEF before symptoms manifest or hospitalization occurs [20,21].

The electrocardiogram (ECG) represents a compelling candidate for this role. The 12-lead ECG is inexpensive, rapid, noninvasive, and routinely obtained across diverse clinical settings—from primary care offices to remote monitoring platforms via smartwatches and consumer-grade wearable devices [22]. In patients with HFpEF, subclinical myocardial electrical remodeling—manifesting as subtle changes in P-wave morphology, QRS duration, ST-T wave abnormalities, and repolarization patterns—may be detectable on the ECG before clinical symptoms emerge. Leveraging artificial intelligence (AI) to extract these patterns from large-scale ECG data could substantially enhance the diagnostic utility of this ubiquitous signal.

There is growing literature demonstrating the utility of ECG-AI for detecting HF subtypes and predicting incident HF. Most published models focus on binary detection of reduced LVEF or treat all HF subtypes as a composite outcome [23,24,25,26]. Fewer studies have specifically targeted HFpEF, and those that have are limited by small samples, single-center designs, or dependence on full 12-lead ECG formats incompatible with wearable deployment [27,28,29,30]. Critically, to our knowledge, no prior study has systematically evaluated the capacity of an ECG-AI model to detect HFpEF at multiple time points preceding clinical diagnosis.

We recently developed and externally validated Wake Forest’s ECG-AI algorithm for Heart Failure—a deep learning model that simultaneously classifies 12-lead (or single Lead I) ECGs into four categories: rEF (LVEF < 40%), mEF (40% ≤ LVEF < 50%), HFpEF, or control [31,32]. In the present study, we apply this validated model to an independent real-world dataset of ECGs recorded prior to clinical HFpEF diagnosis to evaluate risk prediction potential and to quantify the longitudinal association between ECG-AI risk scores and incident HFpEF.

## 2. Materials and Methods

**ECG-AI Model Architecture and Prior Validation**. The ECG-AI model used in this study was developed and validated as described in detail in Karabayir et al. [31]. Briefly, the model employs a modified residual neural network (ResNet) architecture with one-dimensional (1D) convolutional layers, accepting the raw digital waveform from a standard 10 s, 12-lead ECG as input (input shape: 2500 samples × 12 leads after resampling to 250 Hz). The architecture includes multiple residual blocks with batch normalization and dropout regularization, trained using the Adam optimizer with hyperparameter tuning performed on a held-out validation set. A single-lead (Lead I) variant was also developed using an identical procedure [31], enabling deployment on wearable devices with single-lead ECG capability [22].

The model was trained on data from Atrium Health Wake Forest Baptist (AHWFB), Winston-Salem, NC, using over one million ECGs from 165,243 patients split at the patient level into training (80%), internal validation (10%), and holdout (10%) sets. External validation was performed on an independent cohort from the University of Tennessee Health Science Center (UTHSC) in Memphis, TN (72,832 ECGs from 42,880 patients). The 12-lead model achieved AUCs of 0.90, 0.81, and 0.80 for rEF, mEF, and HFpEF, respectively, in the AHWFB holdout and 0.92, 0.76, and 0.73 in the UTHSC external validation cohort [31]. All ECGs used in the present study were drawn exclusively from data not used during model training or primary validation, ensuring independence of the evaluation.

**Study Design and Data Source**. This study used a retrospective observational design, utilizing data from Wake Forest Baptist Health (WFBH), Winston-Salem, NC. The study was conducted under an IRB-approved protocol (IRB No. IRB00079984). As a retrospective secondary analysis of data collected as part of routine clinical care, individual patient informed consent was not required. We adhered to STROBE cohort reporting guidelines.

**HFpEF Case and Control Definition**. HFpEF was defined consistent with our prior validation work [31]: a documented HF diagnosis based on ICD-9/ICD-10 codes in the EHR, combined with an echocardiogram demonstrating LVEF ≥ 50% within ±30 days of the index HF diagnosis date. Patients with a documented HF diagnosis but no available echocardiogram were excluded to prevent misclassification. Control participants were individuals with available ECGs who had no HF diagnosis in the EHR and no recorded LVEF < 50%. For controls, all available ECGs recorded at least six months before the last known clinical encounter were included.

**Cohort Construction and ECG Selection**. ECGs were exported from GE MUSE system (MUSE V9, GE Healthcare, Chicago, IL, USA). Only ECGs recorded prior to the date of clinical HFpEF diagnosis were included for case patients. A single eligible ECG was randomly selected for each patient from available encounters occurring between the earliest ECG and the index HFpEF diagnosis date. The baseline detection window was defined as ECGs recorded within six months prior to HFpEF diagnosis, representing the period closest to clinical recognition. ECGs recorded after the diagnosis date were excluded to avoid post-diagnosis data contamination. For control patients, a single ECG was randomly selected from records with at least six months of subsequent clinical follow-up.

**Assessment of HFpEF Risk Across Prediction Windows**. To evaluate how far in advance the ECG-AI model could detect HFpEF, we analyzed model discrimination at multiple cumulative and fixed-interval time windows before clinical diagnosis. Cumulative windows included case ECGs recorded within 1, 2, 3, 4, 5, and 10 years before diagnosis (each extending from the six-month pre-diagnosis window), while control ECGs were required to be obtained from individuals who remained free of HFpEF throughout the corresponding time window. Fixed-interval windows were also evaluated, including 6 months–1 year, 1–2 years, 2–3 years, 3–4 years, 4–5 years, and 5–10 years before diagnosis. AUC was computed using the model generated HFpEF probability output for each window. Statistical comparisons of AUCs across windows were performed using DeLong’s nonparametric test [33], with a significance threshold of *p* < 0.01.

**Risk Stratification**. The continuous HFpEF probability output was stratified into four ordinal risk groups (Low, Medium, High, and Very High) based on quartile cutoffs established in the validation cohort [31]. The probability thresholds were <0.0038 (Low), 0.0038–0.0096 (Medium), 0.0096–0.0208 (High), and ≥0.0208 (Very High). This approach enables clinically interpretable risk strata for use in downstream survival analyses.

**Comparison With the H2FPEF Score**. For head-to-head comparison with ECG-AI, we utilized the previously described continuous H2FPEF score approach in the subset of patients with complete data for all required components [34]. This approach was selected because it retains continuous predictor information, thereby reducing potential information loss from dichotomization inherent in the original point-based H2FPEF score, and has demonstrated slightly improved discrimination compared with the categorical score [34]. Additionally, the continuous output enables direct comparison of model discrimination using AUC. The continuous H2FPEF score was calculated according to the published approach using age, body mass index, atrial fibrillation, pulmonary artery systolic pressure, and E/e′ ratio. Moreover, this approach was particularly suitable for our cohort because, unlike the original point-based H2FPEF score, it does not require antihypertensive medication data, which were unavailable in our dataset.

**Survival Analysis**. Kaplan–Meier (KM) estimators were used to depict HFpEF-free survival stratified by ECG-AI risk group over a 10-year follow-up period. Nelson–Aalen estimators were used to visualize cumulative hazard by risk group, and log-rank tests were performed to assess between-group differences. For HFpEF cases, time-to-event was defined as the interval from the index ECG date to the date of HFpEF diagnosis. For control participants, follow-up time was censored at the date of the last clinical encounter or, among those who subsequently developed a condition with LVEF < 50%, at the date of the first recorded LVEF < 50%. Cox proportional hazards regression was used to estimate the association between ECG-AI risk group (with Low risk as the reference group) and time to HFpEF diagnosis. Multivariable models were adjusted for age, sex, race (Black or African American), coronary artery disease, diabetes mellitus, myocardial infarction, chronic kidney disease, hyperlipidemia, hypertension, and atrial fibrillation. Model discrimination was evaluated using the concordance index (C-statistic), and the proportional hazards assumption was assessed using Schoenfeld residuals.

All statistical analyses were performed using Python (Python Software Foundation, Wilmington, DE, USA; https://www.python.org/), version 3.13, together with the scikit-learn machine-learning library (https://scikit-learn.org/ [35]), the SciPy scientific computing library (https://scipy.org/ [36]), and the lifelines survival-analysis library (https://lifelines.readthedocs.io/ [37]).

## 3. Results

**Figure 1:**
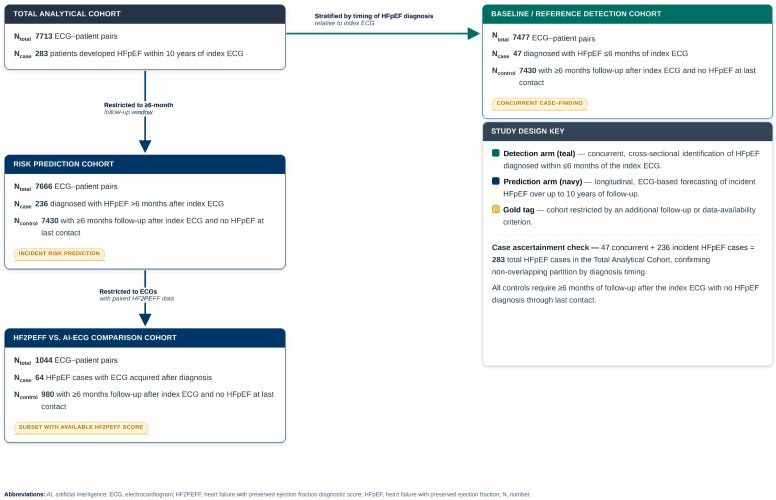
Flow Diagram.

**Cohort Characteristics**. The analytical cohort consisted of 7713 patients with available baseline clinical and electrocardiogram data with baseline characteristics summarized in Table 1. During subsequent follow-up, 283 patients (3.7%) were diagnosed with HFpEF, while 7430 patients (96.3%) did not develop HFpEF. Compared with patients who did not develop HFpEF, those diagnosed with HFpEF were older (66.3 ± 13.6 vs. 60.0 ± 15.7 years, *p* < 0.001) and demonstrated higher right ventricular systolic pressure (RVSP; 40.3 ± 13.4 vs. 32.8 ± 12.2 mmHg, *p* < 0.001). Average E/e′ values were also higher among patients who developed HFpEF (14.8 ± 4.8 vs. 13.4 ± 6.7, *p* = 0.029). Figure 1 shows the overall analytic cohort sample size.

Patients who developed HFpEF had a greater burden of cardiovascular and metabolic comorbidities, including hypertension (73.1% vs. 36.8%, *p* < 0.001), diabetes mellitus (36.0% vs. 16.1%, *p* < 0.001), coronary artery disease (19.1% vs. 6.7%, *p* < 0.001), prior myocardial infarction (21.2% vs. 5.7%, *p* < 0.001), hyperlipidemia (55.8% vs. 26.9%, *p* < 0.001), chronic kidney disease (12.0% vs. 4.1%, *p* < 0.001), and atrial fibrillation (10.2% vs. 2.9%, *p* < 0.001).

There were no significant differences in sex distribution (44.2% vs. 46.2% male, *p* = 0.543), Black race (22.3% vs. 19.7%, *p* = 0.331), body mass index (31.2 ± 8.8 vs. 29.3 ± 12.1 kg/m^2^, *p* = 0.116), smoking status (*p* = 0.090), or obesity prevalence (45.3% vs. 36.5%, *p* = 0.200). Overall, patients who subsequently developed HFpEF represented an older, higher-risk cardiovascular phenotype characterized by increased cardiometabolic disease burdens and elevated markers of cardiac filling pressure. The median follow-up duration was 5.85 years (IQR: 3.93–7.90) for controls and 2.06 years (IQR: 0.80–4.65) for cases (time from index ECG to HFpEF diagnosis).

**ECG-AI Performance Across Prediction Windows.** The ECG-AI model demonstrated consistent discrimination for future HFpEF across all prediction horizons. ECGs obtained within 6 months prior to HFpEF diagnosis yielded an AUC of 0.79 (95% CI: 0.72–0.86), with comparable performance observed across progressively earlier prediction windows, including up to 10 years before diagnosis (AUC range 0.79–0.80). No significant differences in discrimination were observed between the baseline window and any earlier prediction horizon using DeLong’s test (all *p* > 0.01). These findings suggest that ECG-derived markers associated with HFpEF are detectable well before clinical diagnosis and remain stable over time, supporting the potential utility of ECG-AI for long-term HFpEF risk identification. Table 2 summarizes AUC performance, 95% confidence intervals, DeLong *p*-values, and case/control ECG counts for all prediction windows.

**ECG-AI Demonstrates Improved Discrimination Compared with the H2FPEF Score.** To compare the ECG-AI model with the established H2FPEF score, analyses were restricted to patients with complete data required for H2FPEF calculation, including specific echocardiographic parameters and body mass index. Due to these additional data requirements, the comparison cohort was reduced to 1044 patients, of whom 64 subsequently developed HFpEF.

In the near-term prediction window (6 months to 1 year before HFpEF diagnosis), including 9 HFpEF cases and 979 controls, the ECG-AI model demonstrated substantially greater discrimination than the H2FPEF score (AUC 0.83, 95% CI 0.61–0.97 vs. AUC 0.53, 95% CI 0.33–0.72). Given the limited number of events in this early prediction window, longer prediction horizons were also evaluated. For the 5-year cumulative prediction window (6 months to 5 years before diagnosis; 47 HFpEF cases), ECG-AI maintained superior discrimination compared with H2FPEF (AUC 0.77, 95% CI 0.71–0.84 vs. 0.71, 95% CI 0.63–0.79). Similarly, for the 10-year cumulative prediction window (6 months to 10 years before diagnosis), ECG-AI demonstrated an AUC of 0.81 (95% CI 0.72–0.88) compared with 0.74 (95% CI 0.65–0.83) for H2FPEF.

Overall, ECG-AI demonstrated consistently higher or comparable discrimination compared with the H2FPEF score across prediction horizons, including years before clinical HFpEF diagnosis, despite requiring only ECG-derived information.

**Figure 2:**
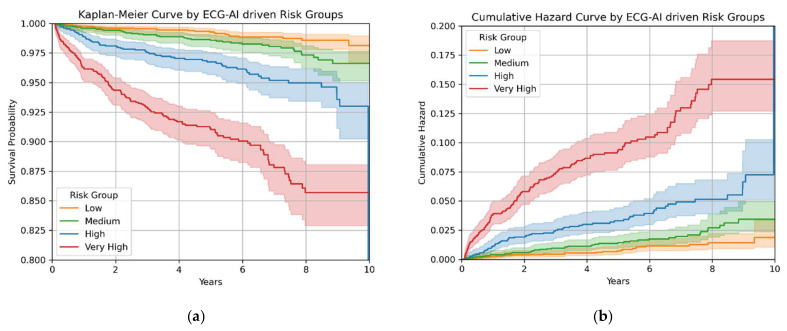
The Kaplan–Meier Survival Curve (**a**) and Nelson–Aalen Cumulative Hazard Curve (**b**) for the ECG-AI Risk Groups for Incident HFpEF.

**Survival Analysis and Risk Stratification.** The ECG-AI–derived risk strata showed clear separation in both Kaplan–Meier and Nelson–Aalen analyses (Figure 2). Participants assigned to the Very High-risk group exhibited the steepest cumulative hazard and shortest HFpEF-free survival, while those in the Low-risk group demonstrated the most favorable event-free trajectory. Log-rank testing confirmed statistically significant differences between all four risk groups (*p* < 0.001).

In Cox proportional hazards analyses, higher ECG-AI–derived HFpEF probability strata were associated with progressively increased risk of incident HFpEF. In the unadjusted model, compared with the lowest probability stratum, increasing ECG-AI probability strata were associated with higher HFpEF risk (HR 1.87, 95% CI 1.13–3.07; HR 4.22, 95% CI 2.64–6.74; and HR 11.18, 95% CI 7.27–17.21 for strata 1–3, respectively; global *p* < 0.001). The unadjusted model demonstrated a C-index of 0.75.

After adjustment for age, sex, race, and baseline cardiovascular comorbidities, the association between ECG-AI probability and incident HFpEF remained robust. Compared with the lowest probability stratum, adjusted hazard ratios were 1.48 (95% CI 0.90–2.44), 2.87 (95% CI 1.79–4.61), and 6.60 (95% CI 4.24–10.28) for increasing probability strata (global *p* < 0.001). The adjusted model demonstrated improved discrimination with a C-index of 0.82.

Within the adjusted model, several established clinical risk factors remained independently associated with HFpEF development, including hypertension (HR 2.39, 95% CI 1.75–3.26; *p* < 0.001), prior myocardial infarction (HR 2.25, 95% CI 1.64–3.09; *p* < 0.001), chronic kidney disease (HR 1.81, 95% CI 1.24–2.63; *p* < 0.001), hyperlipidemia (HR 1.55, 95% CI 1.18–2.04; *p* < 0.001), atrial fibrillation (HR 1.61, 95% CI 1.07–2.40; *p* = 0.02), and older age (HR 1.01 per year increase, 95% CI 1.00–1.02; *p* = 0.03). After accounting for these factors, the ECG-AI HFpEF probability strata remained strongly associated with incident HFpEF.

The proportional hazards assumption was satisfied for all Cox models, with no significant violations identified by Schoenfeld residual testing (all *p* > 0.05). Table 3 summarizes the Cox regression results.

For the adjusted Cox model, we applied the hazard-ratio–based risk score to classify patients into predicted HFpEF and control categories, using the threshold of 1.72378 that maximized the Youden index (0.503); the resulting confusion matrix is shown in Table 4. This analysis included 7666 patients, excluding 47 HFpEF cases whose ECG was recorded within six months of diagnosis. At this threshold the model achieved a sensitivity of 79.2%, specificity of 70.1%, negative predictive value of 99.1%, positive predictive value of 7.9%, and an AUC of 0.807.

## 4. Discussion

**Principal Findings.** This study demonstrates that a deep learning ECG-AI model, trained on real-world EHR data for multiclass HF subtype classification [31], predicted future clinical HFpEF diagnosis from a single ECG per patient, with stable discrimination (AUC 0.78–0.80) across prediction windows extending from six months to ten years before diagnosis and without significant deterioration across progressively earlier windows (all DeLong *p* > 0.01). In a head-to-head comparison restricted to patients with complete data, ECG-AI outperformed the continuous H2FPEF score across every evaluated horizon, improving the AUC by approximately 0.06–0.07 while requiring only ECG-derived information. ECG-AI risk strata were strongly and independently associated with incident HFpEF: relative to the lowest-probability stratum, the highest stratum carried an unadjusted hazard ratio (HR) of 11.18 (95% CI, 7.27–17.21) and an adjusted HR of 6.60 (95% CI, 4.24–10.28) after accounting for age, sex, race, and major cardiovascular and metabolic comorbidities (hypertension, diabetes mellitus, coronary artery disease, prior myocardial infarction, chronic kidney disease, hyperlipidemia, and atrial fibrillation). Adding these clinical covariates to ECG-AI improved the concordance index by 0.07 and 0.09 for the five- and ten-year windows, respectively (adjusted C-index, 0.82). Together, these findings indicate that ECG-derived signatures associated with HFpEF are present years before the syndrome is clinically recognized and carry predictive information beyond that captured by established clinical risk factors and a validated clinical score.

**Prediction, Detection, and Risk Stratification.** An important conceptual point concerns what these analyses do and do not demonstrate. Because HFpEF frequently remains unrecognized for prolonged periods, and because the date of clinical diagnosis does not correspond to the biological onset of disease, our results are most accurately interpreted as prediction of future clinical HFpEF diagnosis and longitudinal risk stratification, rather than detection of disease before its biological onset. The stable discrimination observed from six months to ten years is compatible with two non-mutually exclusive interpretations: the model may capture subclinical electrophysiological changes in evolving HFpEF, or it may identify a high-risk cardiovascular phenotype that precedes and predisposes to the syndrome. Several design choices were made specifically to limit spurious inference—restriction to one ECG per patient to remove intra-subject correlation, adjustment for the full set of major comorbidities, and extension of the analysis to a ten-year horizon. The persistence of strong discrimination at the longest windows argues against the signal being driven solely by occult prevalent disease detected shortly before diagnosis. Nonetheless, in a retrospective design it is not possible to fully disentangle whether ECG-AI reflects true preclinical disease, undiagnosed prevalent disease, or intensified cardiovascular surveillance among higher-risk individuals. Accordingly, the model is best positioned as a prognostic, risk-stratification tool rather than a stand-alone diagnostic test.

**Biological Plausibility and ECG Signatures of Preclinical HFpEF.** The ability of ECG-AI to predict HFpEF years before clinical recognition is biologically plausible and consistent with the emerging understanding of HFpEF as a systemic syndrome driven by chronic inflammation, metabolic dysregulation, and progressive myocardial remodeling [5,7,16]. Structural changes—including cardiomyocyte hypertrophy, interstitial fibrosis, microvascular rarefaction, and altered calcium handling—precede overt symptoms by years and manifest as subtle ECG abnormalities in ventricular repolarization, conduction timing, and P-wave morphology. Explainable-AI analyses of our model using Grad-CAM saliency maps have previously identified QRS-complex prolongation and delayed ventricular activation as the most discriminative features distinguishing HFpEF from controls [31], consistent with known electrophysiological consequences of myocardial fibrosis and impaired diastolic function.

**Comparison with Existing Literature.** Prior ECG-AI work in HF has predominantly focused on reduced LVEF or composite HF endpoints [23,24,25,26]. HFpEF-specific models are comparatively sparse. Kwon et al. [29] achieved an AUC of 0.87 using a 12-lead ECG model with ECG-echocardiogram pairs within a one-week window, limiting real-world generalizability. Hong et al. [30] used a broader ±1-year pairing window and the HFA-PEFF score [11] to define HFpEF cases, achieving an AUC of 0.81 that remained stable at 0.80 even after excluding ECGs within 3 months of echocardiography; however, the HFA-PEFF score has known limitations in sensitivity and specificity [10,12,13,14]. Our work extends this literature in two respects. First, we applied an independently validated multiclass ECG-AI model to a temporal analysis spanning six months to ten years before diagnosis—a horizon not previously reported for HFpEF ECG-AI models. Second, and directly addressing whether the ECG-AI signal adds value beyond information already available in routine practice, we benchmarked the model head-to-head against the continuous H2FPEF score and found consistently superior or comparable discrimination using the ECG alone, despite H2FPEF requiring additional echocardiographic and anthropometric inputs.

**Clinical Implications.** HFpEF remains substantially underdiagnosed, and even among diagnosed patients treatment is often initiated only after significant structural remodeling, recurrent hospitalization, and functional decline [21,38]. A tool that identifies high-risk individuals years in advance could, if prospectively validated, help shift HFpEF care from reactive, diagnosis-triggered management toward proactive, risk-guided evaluation. The most immediate applications are in prioritization rather than autonomous screening: in primary care and multispecialty settings, an ECG-AI risk score computed from a routinely acquired 12-lead ECG could flag patients with HFpEF-predisposing comorbidities (obesity, hypertension, diabetes, atrial fibrillation, chronic kidney disease) for confirmatory echocardiography or natriuretic-peptide testing, and could enrich clinical-trial populations targeting HFpEF prevention [5,21]. Given the low positive predictive value expected at population-level prevalence, ECG-AI is better suited to ruling out low-risk individuals and prioritizing further workup than to establishing a diagnosis on its own. Extension to single-lead and wearable platforms is an appealing future possibility [22] but would require dedicated validation of a single-lead model in this predictive paradigm before any such deployment could be justified.

**Future Research Directions.** Several directions are warranted. First, prospective validation in population-based cohorts in which ECG was collected as part of standardized baseline screening—rather than in response to symptoms—is essential to confirm that the signal reflects true predictive risk rather than clinically indicated surveillance; integration with cohorts such as ARIC, CHS, and JHS would be particularly valuable. Second, adjudication of HFpEF using invasive exercise hemodynamics as the reference standard would strengthen outcome classification beyond ICD-based definitions. Third, the incremental value of ECG-AI over full clinical risk models should be characterized more completely using net reclassification improvement and decision-curve analysis, alongside the discrimination and C-index comparisons reported here. Fourth, multimodal integration—combining ECG-AI with natriuretic peptides, patient-reported symptoms, and wearable physiological signals—may further improve performance. Fifth, single-lead (Lead I) validation is a prerequisite for any wearable application. Finally, evaluation across diverse racial, ethnic, and socioeconomic groups will be critical to ensure equitable deployment.

**Limitations.** This study has several limitations. First, all ECGs were recorded for clinical indications, introducing potential ascertainment and surveillance bias; because the date of clinical diagnosis does not equal disease onset, the results should be read as prediction of future diagnosis rather than detection of biologically preclinical disease, and validation in population-level datasets with standardized ECG collection is needed. Second, HFpEF was defined using ICD codes together with an echocardiographic LVEF ≥ 50% within ±30 days, rather than contemporary diagnostic frameworks such as HFA-PEFF or H2FPEF or objective evidence of elevated filling pressures; applying those frameworks would have required echocardiographic and, ideally, invasive hemodynamic data not uniformly available in routine care. This may result in misclassification in both directions—some labeled cases may not have true HFpEF, and some controls may harbor undiagnosed disease—which could bias performance estimates and, in particular, underestimate specificity. Third, all data originated from a single health system (WFBH), limiting external generalizability. Fourth, medications, device therapies, and comorbidities that independently alter ECG morphology were not modeled. Fifth, the head-to-head H2FPEF comparison was restricted to the subset of patients with complete echocardiographic and anthropometric data (1044 patients, 64 cases), reducing power and potentially selecting a more thoroughly evaluated population. Sixth, although the analysis used one ECG per patient, the modest number of incident cases (283) limits the power for subgroup analyses, and the incremental value beyond full clinical models was not exhaustively characterized (e.g., net reclassification or decision-curve analysis). Seventh, only the 12-lead model was evaluated; assessment of the single-lead (Lead I) model in this predictive paradigm is planned for future work.

## 5. Conclusions

Using a single ECG per patient, an independently validated ECG-AI model predicted incident HFpEF up to ten years before clinical diagnosis, with stable discrimination (AUC 0.78–0.80) across prediction windows and a strong, independent association with future HFpEF in survival analyses (adjusted HR 6.60 for the highest versus lowest risk stratum; adjusted C-index 0.82). ECG-AI outperformed the continuous H2FPEF score across all horizons while requiring only ECG-derived information and added discriminative value beyond established clinical risk factors. These findings support ECG-AI as a low-cost, scalable prognostic and risk-stratification tool that could help prioritize HFpEF workup and enrich prevention trials, rather than as a stand-alone diagnostic test. Prospective validation in community-based cohorts with gold-standard outcome adjudication, characterization of incremental value over full clinical models, and validation of a single-lead variant are the critical next steps before clinical or wearable deployment.

## Figures and Tables

**Table 1:** Baseline Characteristics of the Study Cohort Stratified by HFpEF Status.

Variable		Controls (*n* = 7430)	HFpEF Cases (*n* = 283)	*p*-Value
**Demographics**	Age, years (mean ± SD)	60.0 ± 15.7	66.3 ± 13.6	<0.001
	Male sex, *n* (%)	3432 (46.2%)	125 (44.2%)	0.543
	Black race, *n* (%)	1466 (19.7%)	63 (22.3%)	0.331
	BMI, kg/m^2^ (mean ± SD)	29.3 ± 12.1	31.2 ± 8.8	0.116
**Echocardiographic Parameters**	RVSP, mmHg (mean ± SD)	32.8 ± 12.2	40.3 ± 13.4	<0.001
	E/E′ median (mean ± SD)	13.4 ± 6.7	14.8 ± 4.8	0.029
**Comorbidities**	Chronic kidney disease, *n* (%)	304 (4.1%)	34 (12.0%)	<0.001
	Diabetes mellitus, *n* (%)	1194 (16.1%)	102 (36.0%)	<0.001
	Coronary artery disease, *n* (%)	498 (6.7%)	54 (19.1%)	<0.001
	Myocardial infarction history, *n* (%)	423 (5.7%)	60 (21.2%)	<0.001
	Hyperlipidemia, *n* (%)	1997 (26.9%)	158 (55.8%)	<0.001
	Hypertension, *n* (%)	2731 (36.8%)	207 (73.1%)	<0.001
	Atrial fibrillation, *n* (%)	217 (2.9%)	29 (10.2%)	<0.001
	Obesity, *n* (%)	362 (36.5%)	29 (45.3%)	0.200
**Tobacco use within 1 year**	Never, *n* (%)	803 (47.3%)	21 (35.6%)	0.090
	Passive, *n* (%)	7 (0.4%)	1 (1.7%)	
	Quit, *n* (%)	520 (30.7%)	26 (44.1%)	
	Yes, *n* (%)	353 (20.8%)	10 (16.9%)	

**Table 2:** ECG-AI Model Performance (AUC, 95% CI) Across Prediction Windows Before Clinical HFpEF Diagnosis.

Prediction Window	Sample Size (Case-Control-Total)	AUC (95% CI)	Cumulative Sample Size (Case-Control-Total)	AUC (95% CI)
Baseline (0–6 m)	47-7430-7477	0.793 (0.720–0.862)	47/7430/7457	0.793 (0.720–0.862)
6 m–1 y	44-7413-7457	0.795 (0.721–0.850)	44-7413-7457	0.795 (0.721–0.850)
1–2 y	93-7050-7143	0.784 (0.703–0.839)	93-7050-7143	0.791 (0.740–0.829)
2–3 y	129-6339-6468	0.762 (0.695–0.823)	129-6339-6468	0.789 (0.745–0.820)
3–4 y	154-5501-5655	0.800 (0.704–0.864)	154-5501-5655	0.797 (0.756–0.826)
4–5 y	171-4527-4698	0.662 (0.509–0.787)	171-4527-4698	0.791 (0.758–0.824)
5–10 y	236-899-1135	0.718 (0.647–0.870)	236-899-1135	0.783 (0.749–0.814)

**Table 3:** Cox Proportional Hazards Regression: Association Between ECG-AI Risk Group and Incident HFpEF.

ECG-AI Risk Group	Unadjusted HR (95% CI)	*p*-Value	Adjusted HR (95% CI) *	*p*-Value
Low (Reference)	1.00 (ref)	—	1.00 (ref)	—
Medium	1.87 (1.13–3.07)	0.015	1.48 (0.90–2.44)	0.126
High	4.22 (2.64–6.74)	<0.001	2.87 (1.79–4.61)	<0.001
Very High (per-stratum HR)	11.18 (7.27–17.21)	<0.001	6.60 (4.24–10.28)	<0.001

* Adjusted model includes age, sex, race, coronary artery disease, diabetes mellitus, myocardial infarction, chronic kidney disease, hyperlipidemia, hypertension, and atrial fibrillation.

**Table 4:** Confusion matrix for Cox Model.

True/Predicted	Control	HFpEF	Total	
Control	5236	2194	7430	Specificity = 70.1%
HFpEF	49	187	236	Sensitivity = 79.2%
Total	5285	2381	7666	
	NPV = 99.1%	PPV = 7.9%	AUC = 0.807	<0.001

## Data Availability

The ECG-AI model and evaluation code are available upon reasonable request from the corresponding author. The underlying ECG dataset cannot be publicly released due to IRB restrictions; requests from qualified researchers may be directed to the corresponding author.
